# Supplementary material for: Metabolome Features of COPD: A Scoping Review
Source: Metabolites. 2022 Jul 5;12(7):621. doi: 10.3390/metabo12070621 (PMC9324381; doi:10.3390/metabo12070621)
Supplement: Supplementary file 1 [file metabolites-12-00621-s001.zip › metabolites-1736174-supplementary.pdf]

Supplementary Table S1: Metabolites associated with features of COPD

Outcome	Authors	Title	Sample Description	Analytic Platform	Sample Type	Positively Associated Metabolites	Negatively Associated Metabolites	Pathways Identified
COPD vs Healthy Control	Bertini et al	Phenotyping COPD by 1H NMR metabolomics of exhaled breath condensate	37 COPD/25 controls 67.7% male 70/56 yrs (COPD/HC)	NMR	EBC	lactate; acetate; propionate, serine, proline; tyrosine	acetone; valine; lysine	
COPD vs Healthy Control	Bowerman et al	Disease-associated gut microbiome and metabolome changes in patients with chronic obstructive pulmonary disease	28 COPD/29 HC 39.9% male 67/60 yrs (COPD/HC)	LC-MS	Feces	N-acetyl-cadaverine; N-acetyltaurine; cotinine; N-carboxymethylalanine; asmol	N-acetylglutamate; 6-oxopiperidine-2-carboxylate; N-acetyltaurine; N-acetylproline; gamma-glutamylglutamate; pentadecanoate (15:0); suberate (C8-DC); sebacate (C10-DC); undecanedioate (C11-DC); dodecanedioate (C12); 1-pentadecanoylglycerol (15:0); 2-palmitoyl-galactosylglycerol (16:0)*; oleanolate; harmane; N-carbamoylglutamate	
COPD vs Healthy Control	Bregy et al	Real-time mass spectrometric identification of metabolites characteristic of chronic obstructive pulmonary disease in exhaled breath	22 COPD/14 controls 61.1% male 58.6/58.1 yrs (COPD/HC)	SESI-HRMS	EBC	2-hydroxyisobutyric acid; Aspartic acid semialdehyde; Acetohydroxybutanoic acid; 2-oxoglutaric acid semialdehyde	Pyridine; 11-hydroxyundecanoic acid; (+)-γ-hydroxy-L-homoarginine; Oxo-tetradecenoic acid; Hexadecatrienoic acid; Oxo-heptadecanoic acid	
COPD vs Healthy Control	Callejon-Leblic et al	Study of the metabolomic relationship between lung cancer and chronic obstructive pulmonary disease based on direct infusion mass spectrometry	30 COPD/30 HC/30 Lung Cancer 71.1% male 67/56/66 yrs (LC/HC/COPD)	DI-ESI-QTOF-MS	Serum	Acetic acid; Adenine; Dopamine; Phenylalanine; Arginine; Tyrosine; Phosphocholine; Glucose; Acetyl-Carnitine; Propyonyl-Carnitine; Palmitoleic acid; Adenosine; Hydroxyhexanoylcarnitine; Oleic acid; Glycerophosphocholine; LPC(16:0); LPC(18:3); LPC(18:1); LPC(18:0); LPC(20:4); LPC(20:2); PC(16:0/16:1); PC(16:0/18:0); PC(16:0/20:5); PC(18:2/18:2); PC(18:2/18:1); PC(18:2/18:0); PC(16:0/22:6); PC(18:1/20:4); TAG(16:0/16:0/18:1); TAG(16:0/16:0/18:0); TAG(16:0/18:3/18:2); TAG(16:0/18:1/18:1)	Pyroglutamate; Aspartic acid; Creatine; Ornithine; Glutathione; Docosahexanoic acid; DAG(18:3/22:6); DAG(18:2/22:6)	
COPD vs Healthy Control	Cazzola et al	Analysis of exhaled breath fingerprints and volatile organic compounds in COPD	27 COPD/7 HC 79.4% male 72/27 yrs (COPD/HC)	Enose GC-MS	EBC	Decane; 6-ethyl-2-methyl-Decane	1,3,5-tri-tert-butyl-Benzene; Butylated hydroxytoluene; 3-ethyl-4-methyl-Hexane; Hexyl ethylphosphonofluoridate; Limonene; 2,4,4-trimethyl-1-Pentene; 2-Propanol	
COPD vs Healthy Control	Celejewska-Wójcik et al.	Eicosanoids and Eosinophilic Inflammation of Airways in Stable COPD	76 COPD/37 HC 68% male 65 years (mean age)	GC-MS HPLC-MS	Sputum	LTE4; LTD4; PGE2; PGD2; 8-izo-PGE2; 8-izo-PGF2a; 5-oxo-ETE; 12-oxo-ETE; 11-dehydro-TBX2	Tetranor-PGE-M; Tetranor-PGD-M	

Outcome	Authors	Title	Sample Description	Analytic Platform	Sample Type	Positively Associated Metabolites	Negatively Associated Metabolites	Pathways Identified
COPD vs Healthy Control	Chen et. al.	Serum Metabolite Biomarkers Discriminate Healthy Smokers from COPD Smokers	41 COPD Smoker/37 Healthy Smoker /37 Healthy Non-Smoker 78% male 39.5/41.8/53.2 yrs (HC/Smoker/COPD)	LC-MS	Fasting Serum	Cotinine; 3-Hydroxycotinine; Unknown 1; Quinic acid; PI(32:2); PI(32:1); 5-Acetylamino-6-formylamino-3-methyluracil; Unknown2; PI(32:0); PE(35:1); Unknown3; 1α,25-dihydroxy-23-thiavitamin D3; PI(40:4); Unknown; 6-methyltetrahydropterin; PE(33:1); PI(34:1)		
COPD vs Healthy Control	Esther et al	Identification of Sputum Biomarkers Predictive of Pulmonary Exacerbations in Chronic Obstructive Pulmonary Disease	SPIROMICS cohort 77 HC/341 smokers perserved spirometry/562 COPD 53% male 55.4/59.6/65 yrs (HC/Smokers/COPD)	UPLC-MS	Sputum	Sialic Acid; Hypoxanthine; Xanthine; Methylthioadenosine; Adenine; Glutathione		
COPD vs Healthy Control	Gillenwater et al	Metabolomic Profiling Reveals Sex Specific Associations with Chronic Obstructive Pulmonary Disease and Emphysema	COPDGene Cohort: n=839; 51.7% male; 67 yrs SPIROMICS Cohort: n=446; 52% male; 64.5 yrs	LC-MS (Metabolon)	Plasma	*Network Module (female)*: Ceramides, Sphingomyelins *Network Module (Male)*: Steroids (Androgenic, Pregnenolone, Corticosteroids, Progestin)) *Network Module (Male & COPD)*: Ceramides, Sphingomyelins *Males*: ceramide (d18:1/17:0, d17:1/18:0); octadecenedioate (C18:1-DC); N-stearoyl-sphingosine (d18:1/18:0) *Males & Females*: 4 acyl carnitines.	*Network Module (COPD)*: Diacylglycerols, Phosphatidylethanolamines (PE), Acyl Carnitines *Network Module (COPD)*: Amino Acids (Gamma-glutamyl Amino Acid, Glutamate Metabolism, Branched Chain Amino Acids, Urea cycle; Arginine and Proline Metabolism, Lysine Metabolism, Methionine, Cysteine, SAM and Taurine Metabolism, Phenylalanine Metabolism), Bile Acids, Acyl Cholines, Lysophospholipids) *Males & Females*: retinol (Vitamin A); phosphocholine; ergothionene; 3-formylindole *Male & COPD*: sphingomyelins *Female & COPD*: phosphatidylethanolamines; acyl carnitines *Opposite Direction By Sex*: ceramide (d18:1/17:0, d17:1/18:0)	
COPD vs Healthy Control†	Kilk et al	Phenotyping of Chronic Obstructive Pulmonary Disease Based on the Integration of Metabolomes and Clinical Characteristics	25 COPD; 21 HC 73.9% male 67/37 yrs (COPD/HC)	Untargeted: Electrospray ionization MS Targeted: HPLC-MS	Serum	Ala; Arg; Gln; Orn; Phe; Tyr; Kynurenine; phosphatidylcholine aa C38:5; Trans 4-OH Pro; lysoPC a C16:0; lysoPC a C18:1; lysoPC a C18:2; lysoPC a C20:3; lysoPC a C17:0	aceylcarnitine C10; aceylcarnitine C16:1; aceylcarnitine C18; Ser; lysoPC a C18:0; phosphatidylcholine aa C32:2; phosphatidylcholine aa C34:2; phosphatidylcholine aa C34:4; phosphatidylcholine aa C36:4; phosphatidylcholine aa C36:6; phosphatidylcholine aa C38:6; phosphatidylcholine aa C42:5; phosphatidylcholine ae C34:3; phosphatidylcholine ae C36:4; phosphatidylcholine ae C38:0; hydroxylated sphingomyeline C14:1; hydroxylated	

Outcome	Authors	Title	Sample Description	Analytic Platform	Sample Type	Positively Associated Metabolites	Negatively Associated Metabolites	Pathways Identified
							sphingomyeline C16:1; hydroxylated sphingomyeline C22:1; hydroxylated sphingomyeline C22:2; hydroxylated sphingomyeline C24:1	
COPD vs Healthy Control†	Kim et al	Metabolic Fingerprinting Uncovers the Distinction Between the Phenotypes of Tuberculosis Associated COPD and Smoking-Induced COPD	59 T-COPD (TB)/70 S-COPD 39 healthy controls 100% male 66/68/55 yrs (TB/COPD/HC)	LC-QTOF-MS LC-MS/MS	Plasma Urine	Acylcarnitines C12; Acylcarnitines C141; Acylcarnitines C161; Acylcarnitines C10; Acylcarnitines C121; Acylcarnitines C8; Acylcarnitines C14; Acylcarnitines C16; Acylcarnitines C181; Acylcarnitines C142; Acylcarnitines C7-DC; Acylcarnitines C101; Acylcarnitines C182; Acylcarnitines C2; PC ae C425; Acylcarnitines C6 C41-DC; Glu; PC ae C405; PC aa C321; PC ae C424; Acylcarnitines C9	lysoPC a C182; H1; PC aa C342; Pro; alpha-AAA; PC aa C362; Ala; C31; Inosine	
COPD vs Healthy Control	Liu et al	Identification of lipid biomarker from serum in patients with COPD	20 COPD/5 Control 60% males 65.8/69.3 yrs (COPD/HC)	ESI-MS	Serum	C16:E1; TAG(54:6); PC(32:1); SM(22:0); ePS(40:5); PC(40:4); PE(34:1); PE(38:0); PE(40:5); PE(44:12) *Ratios*: C16:1 CE/C19:0 CE; PC(40:4)/ePC(38:2)	: ePE(34:2); ePS(38:3); LPE(20:2); PI(36:6); PI(44:6); TAG(54:5); ePE(40:3) *Ratios*: PI(38:4)/C16:1 CE; PI(36:2)/C16:1 CE; ePC(38:2)/C16:1 CE; LPC(18:0)/C20:3 CE; LPC(16:1)/C16:1 CE; PC(32:0)/C16:1 CE; PC(34:3)/C16:1 CE; PC(38:1)/C16:1 CE	
COPD vs Healthy Control	Prokić et al	A cross-omics integrative study of metabolic signatures of chronic obstructive pulmonary disease	*Discovery*: 4948 Rotterdam Study (44.2% male; 70.3 yrs); 609 Erasmus Rucphen Family study (44.2% male; 49 yrs) *Validation*: 717 Lifelines-DEEP study (43.7% male; 46 yrs); 11,498 FINRISK (47.7% male 49.7 yrs); 854 Prospective Investigation of the Vasculature in Uppsala Seniors (51.8% male; 70 yrs)	NMR	Fasting Plasma	*Discovery*: GlycA; 3-hydroxybutyrate; Free cholesterol in med. HDL; Acetoacetate *Validation*: GlycA	Discovery: Histidine; Acetoacetate	
COPD vs Healthy Control	Rodríguez-Aguilar et al	Ultrafast gas chromatography coupled to electronic nose to identify volatile biomarkers in exhaled breath from chronic obstructive	23 COPD/33 HC 48.2% male 67.7/55.6 yrs (COPD/HC)	eNose (ultrafast GC)	Fasting Exhaled Breathe	Alpha-pinene; Acetaldehyde; 2-Butyl octanol; Octane; Methyl isobutyrate; Butanal; 2-Propanol; 3-Hexanone; 3-methyl-Cyclopentanone; Propanal	Delta-dodecalactone; 2-Methylbutanoic acid; Indole; 2-Acetylpyridine; Tetradecane; [E]-Cinnamaldehyde; Vinylpyrazine	

Outcome	Authors	Title	Sample Description	Analytic Platform	Sample Type	Positively Associated Metabolites	Negatively Associated Metabolites	Pathways Identified
		pulmonary disease patients: A pilot study						
COPD vs Healthy Control	Titz et al	Alterations in Serum Polyunsaturated Fatty Acids and Eicosanoids in Patients with Mild to Moderate Chronic Obstructive Pulmonary Disease (COPD)	39 Never Smokers/39 Current Smokers/39 COPD 38 Former Smokers 55.5% males 55.8/55.5/57.9/57.1 yrs (NS/S/COPD/FS)	4 MS platforms: shotgun; triacylglycerol; ceramide and cerebroside; eicosanoid lipidomics;	Serum	CE16:1; Cer(d18:1/22:1); DAG 18:1/18:1; DAG 16:0/18:1; 15-HETrE; 14,15-DHET; 11,12-DHET; PC16:1/18:1; PC16:0/18:1; PC16:0/16:1; PE 18:1/18:1; PE 18:0/20:4; PE 16:0/20:4: TAG 54:3 total(16:0/18:1/20:2)(18:0/18:1/18:2)(18:1/18:1/18:1); TAG 54:2 total(16:0/18:1/20:1)(18:0/18:1/18:1); TAG 52:3 total(16:0/18:1/18:2)(16:1/18:1/18:1); TAG 52:2 total(16:0/18:0/18:2)(16:0/18:1/18:1); TAG 52:1 total(16:0/18:0/18:1); TAG 51:2 total (15:0/18:1/18:1) *Clusters*: (HODE); (CE; PC; LPC/LPE [x/20:5]; DHA/EPA)		triacylglycerols (TAGs), diacylglycerols (DAGs), and phosphatidylethanolamines (PEs)
COPD vs Healthy Control	Wang et al	Metabonomic Profiling of Serum and Urine by 1H NMRBased Spectroscopy Discriminates Patients with Chronic Obstructive Pulmonary Disease and Healthy Individuals	32 COPD/21 HC 56.6% male 71/63 yrs (COPD/HC)	NMR	Serum	Glycerolphosphocholine	Alanine; Isoleucine; CH3-(CH2)n-HDL; Leucine	
COPD vs Healthy Control	Wang et al	Metabonomic Profiling of Serum and Urine by 1H NMRBased Spectroscopy Discriminates Patients with Chronic Obstructive Pulmonary Disease and Healthy Individuals	32 COPD/21 HC 56.6% male 71/63 yrs (COPD/HC)	NMR	Urine	Acetate; Acetoacetate; Acetone; Carnosine; m-Hydroxyphenylacetate; Phenylacetyglycine; Pyruvate; alpha-Ketoglutarate	1-methylnicotinamide; Creatinine; Lactate	
COPD vs Healthy Control	Westhoff et al	Differentiation of chronic obstructive pulmonary disease (COPD) including lung cancer from healthy control group by breath analysis using ion mobility spectrometry	97 COPD (35 w/Bronchial Carcinoma; 62 w/o)/35 HC *No other details provided*	IMS-MCC	Exhaled Breathe	cyclohexanone		

Outcome	Authors	Title	Sample Description	Analytic Platform	Sample Type	Positively Associated Metabolites	Negatively Associated Metabolites	Pathways Identified
COPD vs Healthy Control	Zheng et al	Predictive diagnosis of chronic obstructive pulmonary disease using serum metabolic biomarkers and least-squares support vector machine	54 COPD/74 HC 60.9% male 71.3/65.1 yrs (COPD/HC)	NMR	Fasting Serum	formate	N-acetyl-glycoprotein, lipoproteins mainly including LDL and VLDL, pUFA, glucose, alanine , leucine, histidine, valine, and lactate	
COPD vs Healthy Control†	Zhou et al	Plasma Metabolomics and Lipidomics Reveal Perturbed Metabolites in Different Disease Stages of Chronic Obstructive Pulmonary Disease	48 HC/48 Stable COPD/48 AECOPD 79.2% male 63.7/67.3/66.2 yrs (HC/S-COPD/AECOPD)	LC-MS	Fasting Plasma	Xanthine; Dimethylglycine; Phenylalanine; D-Alanyl-D-alanine; Cysteinylglycine; N-Acetylputrescine; 2-Methylglutaric acid; N-Formyl-L-methionine; Nicotinic acid; Pyruvic acid; 4-Acetamidobutanoic acid; L-Threonine; 2-Hydroxbutyric acid; Glutamylglutamine; Sorbitol; Cafestol; L-Gulonolactone; Xanthopurpurin; Alph-ketoisovale; caffeic acid	Leucine; Oxazepam; L-Tryptopha; Serotonin; gama-Glutamylleucine; Adenosine monophos; 3'-AMP; 3-Hydroxyflavone; Glyceric acid; ADP; Creatinine; Xanthine; Choline; 2-Hydroxyphenethyl; Maltotriose; L-Lysine; Gabapentin; Pyroglutamic acid; trans-Cinnamic acid	Aminoacyl-tRNA biosynthesis; Nitrogen metabolism; valine, leucine and isoleucine biosynthesis; arginine and proline metabolism; glycerine, serine, and threonine metabolism; phenylalanine metabolism; Pantothenate and CoA biosynthesis; Beta-alanine metabolism
COPD (non-survivors) vs Healthy Control†	Pinto-Plata et al	Plasma metaoblomics and clinical predictors of survival differences in COPD patients	90 COPD/30 Controls 66% males 68.7/68 yrs (COPD/HC)	LC-MS GC-MS	Plasma	2-ethylhexanoate; bradykinin, des-arg(9); Hexadecanedioate; Fucose; HWESASXX*; Malate; 1-arachidonoylglycerophosphoinositol*; dihomo-linolenate (20:3n3 or n6); Succinate; Fumarate; Lactate; Aspartate; Ornithine; 2-hydroxypalmitate; Leucylleucine; arachidonate (20:4n6); Glycerate; Tetradecanedioate; C-glycosyltryptophan	Benzoate; 2-aminobutyrate; dehydroisoandrosterone sulfate (DHEA-S); caproate (6:0); Isovalerate; Nicotinamide; epiandrosterone sulfate; gamma-CEHC; 1-linoleoylglycerophosphocholine (18:2n6); 2-linoleoylglycerophosphocholine*; Piperine; Alanine; Methionine; androsterone sulfate; gamma-tocopherol; Tryptophan; Indoleacetate; Creatinine; pregnenolone sulfate; Valine	*COPD Survivors vs Non-Survivors:* Glyoxylate and dicarboxylate metabolism; Citrate cycle (TCA cycle)
COPD vs control†	Yu et al	Metabolomics Identifies Novel Blood Biomarkers of Pulmonary Function and COPD in the General Population	ARIC Cohort (n= 2,354 African Americans, 1529 European American); 39.8% male; 53.0/54.6 yrs (AA/EA) KORA cohort (n=859 Europeans); 46.8% male; 53.8 yrs	LC-MS	Serum	3-(4-hydroxyphenyl)lactate; 3-methoxytyrosine; homocitrulline; ornithine; succinylcarnitine; oleoylcarnitine; 5-dodecenoate (12:1n7); 7-alpha-hydroxy-3-oxo-4-cholestenoate (7-Hoca); glycerol; pseudouridine; theophylline; 1-methylurate	serotonin (5HT); glycerate; docosahexaenoate (DHA, 22:6n3); androsterone sulfate	
COPD vs Control Severity	Ubhi et al	Metabolic profiling detects biomarkers of protein degradation in COPD patients	ECLIPSE Cohort 15 Nonsmokers/53 Smoker/163 COPD 64.5% males 61/57/64.2 (NS/S/COPD)	NMR; LC-MS	Fasting Serum	COPD: 3-methylhistidine; Glutamine; Phenylalanine; 3-hydroxybutyrate; Acetoacetate; Ascorbate GOLD IV: Trimethylamine; 3-methylhistidine; Glutamine; Phenylalanine; 3-hydroxybutyrate; Ascorbate	COPD: N,N-dimethylglycine; LDL/VLDL; Polyunsaturated lipid; O-acetylated glycoproteins GOLD IV: N,N-dimethylglycine; 3-hydroxyisobutyrate; Isobutyrate; Isoleucine; Valine; Methionine; HDL; LDL/VLDL; Polyunsaturated lipid; Glycerol	

Outcome	Authors	Title	Sample Description	Analytic Platform	Sample Type	Positively Associated Metabolites	Negatively Associated Metabolites	Pathways Identified
COPD vs Healthy Control (Severity)	Xue et al	Metabolomic profiling of anaerobic and aerobic energy metabolic pathways in chronic obstructive pulmonary disease	140 COPD/20 HC 78.8% males 60/52 yrs (COPD/HC)	UHPLC-Q-TOF/MS	Fasting Serum	*GOLD IV*: pyruvate; lactic acid	TCA Cycle	
COPD vs Smokers	Berdyshev et al	Ceramide and sphingosine-1 phosphate in COPD lungs	69 COPD/16 Smoker without COPD/13 Interstitial lung disease 48.4% males 66/62.8/60.5 years (Smoker/COPD/ILD)	LC-MS	Lung tissue	ceramides (GOLD0 - 2) sphingosine-1 phosphate	ceramides (GOLD 3-4)	ceramide-to-S1P metabolism controlled by sphingosine kinase-1 (SphK1)
COPD vs Smokers	Diao et al	Disruption of histidine and energy homeostasis in chronic obstructive pulmonary disease	79 COPD/59 smokers no COPD/7 non-smokers 100% male 58.8/56.8/57.4 yrs (COPD/Smoker/NS)	NMR	Fasting Serum and Plasma	histamine	creatine; glycine; histidine; threonine	
COPD vs Smokers	Gaida et al	A dual center study to compare breath volatile organic compounds from smokers and non-smokers with and without COPD	52 Healthy non/ex smoker/52 COPD non/ex smoker/29 Healthy Smoker/ 37 COPD Smoker 52% male 35/64/43.5/61 yrs (HC/COPD NS/HS/COPD Smoker)	Enose IMS detector GC-IMS TD-GC-APCI-MS	Exhaled Breathe	Indole; 1,6-Dimethyl-1,3,5-heptatriene; m,p-Xylene; 1-Ethyl-3-methyl benzene; Toluene; Benzene		
COPD vs Smokers	Naz et al	Metabolomics analysis identifies sex-associated metabotypes of oxidative stress and the autotaxin–lysoPA axis in COPD	Karolinska COSMIC cohort 38 Never-smokers/40 smokers/38 COPD 51.7% males 58.8/53/60.3 yrs (NS/Smokers/COPD)	LC-MS	Serum	*Both Sexes*: 12-HETE; 4-HDoHE; Carnitine(C12:0); Carnitine(C14:0); Carnitine(C14:1); Carnitine(C16:1); Carnitine(C18:1); Carnitine(C18:2); Dityrosine; Erythronicacid; Hypoxanthine; Inosine; Leu-Pro; LysoPA(16:0); LysoPA(18:2); LysoPC(15:0); Malate; N-Oleoyl-L-serine; OH-hexadecanoic acid; Palmitoleic Acid; Phenylpropionylglycine; S-1P; Succinate Females: 12-HETE; 4-HDoHE; Carnitine(C12:0); Dityrosine; Erythronicacid; LysoPA(16:0); LysoPA(18:2); LysoPC(15:0); Malate; Myristoylglycine; N-Oleoyl-L-serine;	*Both Sexes*: Leu-Pro; PC(16:1/P-18:1)	*Both Sexes*: Citrate (tricarboxylic acid) cycle; Glycerophospholipid metabolism; Pyruvate metabolism *Females*: Fatty acid biosynthesis; Sphingolipid metabolism *Males*: cAMP signalling pathway; Retrograde endocannabinoid signalling; Tryptophan metabolism

Outcome	Authors	Title	Sample Description	Analytic Platform	Sample Type	Positively Associated Metabolites	Negatively Associated Metabolites	Pathways Identified
						Phenylpropionylglycine; S-1P; Sn-1P; Succinate		
Case vs Control (emphysema)	Ubhi et al	Metabolic profiling detects biomarkers of protein degradation in COPD patients	ECLIPSE Cohort 41 Non-emphysematous/ 77 Emphysematous 68.6% males 64/64 yrs (Non/Emph)	NMR; LC-MS	Fasting Serum	3-methylhistidine; Glutamine; Phenylalanine; 3-hydroxybutyrate	Creatine; Glycine; N,N-dimethylglycine; 3-hydroxyisobutyrate; Isoleucine; Leucine; Valine; HDL; LDL/VLDL; Polyunsaturated lipid; Monoglyceride; Glycerol; O-acetylated glycoproteins; N-acetylated glycoproteins	
Case vs Control (GOLD)	Ubhi et al	Targeted metabolomics identifies perturbations in amino acid metabolism that sub-classify patients with COPD	ECLIPSE Cohort 30 smoker/30 COPD/100% male 57/65 (smoker/COPD)	LC-MS/MS	Fasting Serum	Beta-Aminoisobutyric acid*; 3-Methylhistidine; Aspartic acid; 1-Methylhistidine; Glutamine; gamma-Aminobutyric acid; Arginine	alpha-Aminobutyric acid*; Proline; Aminoadipic acid; 4-Hydroxyproline; Leucine; Valine; Isoleucine	
Case vs Control (emphysema)	Ubhi et al	Targeted metabolomics identifies perturbations in amino acid metabolism that sub-classify patients with COPD	ECLIPSE Cohort 21 no emphysema/38 Emphysema 100% male 65/64 yrs (noEmph/Emph)	LC-MS/MS	Fasting Serum	Glutamine; Aspartic acid; 3-Methylhistidine; 1-Methylhistidine; Histidine; Arginine; Serine; Proline	Tryptophan; Sarcosine; beta-Aminoisobutyric acid; Aminoadipic acid	
Case vs Control (cachexic)	Ubhi et al	Targeted metabolomics identifies perturbations in amino acid metabolism that sub-classify patients with COPD	ECLIPSE Cohort 30 no Cachexia/29 Cachexia 100% male 64/61 yrs (no Cach/Cach)	LC-MS/MS	Fasting Serum	Serine; Glutamine; Glutamic acid; Histidine; Asparagine; Aspartic acid; Proline; Arginine; Phenylalanine	Cystathionine; Thiaproline; 1-Methylhistidine; Sarcosine; beta-Aminoisobutyric acid; 4-Hydroxyproline; Hydroxylysine; Tryptophan; Aminoadipic acid	
Emphysema	Bowler et al	Plasma Sphingolipids Associated with Chronic Obstructive Pulmonary Disease Phenotypes	COPDGene Cohort **Targeted: n**=129; 57% male; 63 yrs **Untargeted: n**=131; 56% male; 64 yrs	LC-MS	Plasma		Ganglioside GM3 (d18:1/16:0); Sphingomyelin(d18:0/24:1(15Z)); Sphingomyelin(d18:1/14:0); Sphingomyelin(d18:1/16:0); Sphingomyelin(d18:1/16:1); Sphingomyelin(d18:1/24:1(15Z)) Sphingomyelin(d18:2/14:0)	
Emphysema	Halper-Stromberg et al	Bronchoalveolar Lavage Fluid from COPD Patients Reveals More Compounds Associated with Disease than Matched Plasma	SPIROMICS cohort 12 Non-smokers/56 Smokers/47 COPD 50.7% male 56/58/64 yrs (NS/Smokers/COPD)	LC-MS	BALF	leucine; lysine		amino acid derived compounds; fatty acids; phospholipids (phosphatidylethanolamines, phosphatidylinositols, phosphatidylcholines), carnitines
Emphysema	Labaki et al	Serum amino acid concentrations and	SPIROMICS cohort: n=157;	NMR	Serum		tryptophan	

Outcome	Authors	Title	Sample Description	Analytic Platform	Sample Type	Positively Associated Metabolites	Negatively Associated Metabolites	Pathways Identified
		clinical outcomes in smokers	49.7% male; 53.7 yrs					
Emphysema	Mastej et al	Identifying Protein–metabolite Networks Associated with COPD Phenotypes	COPDGene Cohort 426 Controls/478 COPD/92 PRISm 12 missing Spirometry 50.9% males 64.6/71.1/67.3/72 yrs (HC/COPD/PRISm/MissSpiro)	LC-MS	Plasma		1-stearoyl-2-linoleoyl-GPI (18:0/18:2); androsterone glucuronide; 1-stearoyl-2-docosahexaenoyl-GPE (18:0/22:6); 1-palmitoyl-2-docosahexaenoyl-GPE (16:0/22:6); 1-palmitoyl-2-linoleoyl-GPI (16:0/18:2); 1-ribosyl-imidazoleacetate; Valine; palmitoyl-linoleoyl-glycerol (16:0/18:2) [2]; 1-stearoyl-2-arachidonoyl-GPI (18:0/20:4); Glutamate	
Emphysema	Gillenwater et al	Metabolomic Profiling Reveals Sex Specific Associations with Chronic Obstructive Pulmonary Disease and Emphysema	COPDGene Cohort: n=839; 51.7% male; 67 yrs SPIROMICS Cohort: n=446; 52% male; 64.5 yrs	LC-MS (Metabolon)	Plasma	*Network Module*: Amino Acids, Bile Acids, Acyl Cholines, Lysophospholipids *Network Module*: Sterioids *Network Module*: Xenobiotics, Amino Acids, and TCA cyclebrown (Amino Acids, Bile Acids, Acyl Cholines, Lysophospholipids) *Network Module*: Steroids	*Full Cohort*: 5-hydroxylysine, isovalerate (C5), X-17357 *Males*: 5-hydroxylysine; X-17357 *Females*: 2,3-dihydroxy-2-methylbutyrate; alpha-ketoglutaramate; homocitrulline	
Emphysema	Gillenwater et al	Plasma Metabolomic Signatures of Chronic Obstructive Pulmonary Disease and the Impact of Genetic Variants on Phenotype-Driven Modules	Discovery - COPDGene Cohort: n=957; 51.2% male; 68.3 yrs Replication - COPDGene-Emory: n=271; 46.9% male; 67.3 yrs Replication - SPIROMICS-Metabolon: n=445; 54.8% males; 65.3 yrs Replication - SPIROMICS-UC: n=76; 52.6% male; 61.6 yrs	LC-MS (Metabolon)	Plasma	tricarboxylic cycle metabolite (citrate)		
Emphysema	Diao et al	Disruption of histidine and energy homeostasis in chronic obstructive pulmonary disease	79 COPD/59 smokers no COPD/7 non-smokers 100% male 58.8/56.8/57.4 yrs (COPD/Smoker/NS)	NMR	Fasting Serum and Plasma	creatine; histidine; 3-hydroxybutyrate; betaine; carnitine; glutamine; acetylcarnitine; valine		

Outcome	Authors	Title	Sample Description	Analytic Platform	Sample Type	Positively Associated Metabolites	Negatively Associated Metabolites	Pathways Identified
FEV1 (% predicted) FEV1/FVC	Bregy et al	Real-time mass spectrometric identification of metabolites characteristic of chronic obstructive pulmonary disease in exhaled breath	22 COPD/14 controls 61.1% male 58.6/58.1 yrs (COPD/HC)	SESI-HRMS	EBC	Pyridine; 11-hydroxyundecanoic acid; (+)-γ-hydroxy-L-homoarginine; Oxo-tetradecenoic acid; Hexadecatrienoic acid; Oxo-heptadecanoic acid	2-hydroxyisobutyric acid; Aspartic acid semialdehyde; Acetohydroxybutanoic acid; 2-oxoglutaric acid semialdehyde	
FEV1 FEV1/FVC	Celejewska-Wójcik et al.	Eicosanoids and Eosinophilic Inflammation of Airways in Stable COPD	76/37 COPD/HC 68% male 65 years (mean age)	GC-MS HPLC-MS	Sputum		PGD2; 11-dehydro-TBX2	
FEV1 % predicted FEV1/FVC	Cruickshank-Quinn et al.	Metabolomics and transcriptomics pathway approach reveals outcome-specific perturbations in COPD	COPDGene Cohort: (n=149); 53.0% male; 63.1 yrs	LC-MS	Plasma	PE(P-38:2); Sphinganine-1-phosphate; Eicosapentaenoyl PAF C-16; Purine	cis-7-Hexadecenoic acid methyl ester; LysoPC(16:0); Ceramide (d18:1/24:1); Octanoyl-L-carnitine; Glucosaminic acid; Oleamide; DG(34:1); LysoPC(18:1); PC(36:2); PC(36:4); PC(36:5); N-Acetylserotonin; Glutathione; Uridine; Cortisone	Endocytosis; Fc gamma R-mediated phagocytosis; Glycerophospholipid metabolism; Hippo signaling pathway; Jak-STAT signaling pathway; Lysosome; mTOR signaling pathway; Neurotrophin signaling pathway; NF-kappa B signaling pathway; Notch signaling pathway; Osteoclast differentiation; Peroxisome; Phagosome; Phosphatidylinositol signaling system; Primary immunodeficiency; Ribosome; SNARE interactions in vesicular transport; Sphingolipid metabolism; Sphingolipid signaling pathway; T cell receptor signaling pathway; Th1 and Th2 cell differentiation; Th17 cell differentiation; Autophagy; Fat digestion and absorption; Glycerolipid metabolism; Hematopoietic cell lineage
FEV1/FVC	Yu et al	Metabolomics Identifies Novel Blood Biomarkers of Pulmonary Function and COPD in the General Population	ARIC Cohort (n= 2,354 African Americans, 1529 European American); 39.8% male; 53.0/54.6 yrs (AA/EA) KORA cohort (n=859 Europeans); 46.8% male; 53.8 yrs	LC-MS	Serum	androsterone sulfate; dehydroisoandrosterone sulfate (DHEA-S); lathosterol	3-methoxytyrosine; glycerol; oleoylcarnitine; 7-alpha-hydroxy-3-oxo-4-cholestenoate (7-Hoca); theophylline	
FEV1/FVC	Prokić et al	A cross-omics integrative study of metabolic signatures of chronic obstructive pulmonary disease	**Discovery**: 4948 Rotterdam Study (44.2% male; 70.3 yrs); 609 Erasmus Rucphen Family study (44.2% male; 49 yrs) **Validation**: 717 Lifelines-DEEP study (43.7%	NMR	Fasting Plasma	*Discovery*: Valine	*Discovery*: GlycA	

Outcome	Authors	Title	Sample Description	Analytic Platform	Sample Type	Positively Associated Metabolites	Negatively Associated Metabolites	Pathways Identified
			male; 46 yrs); 11,498 FINRISK (47.7% male 49.7 yrs); 854 Prospective Investigation of the Vasculature in Uppsala Seniors (51.8% male; 70 yrs)					
FEV1/FVC - post†	Gillenwater et al	Plasma Metabolomic Signatures of Chronic Obstructive Pulmonary Disease and the Impact of Genetic Variants on Phenotype-Driven Modules	Discovery - COPDGene Cohort: n=957; 51.2% male; 68.3 yrs<br>Replication - COPDGene-Emory: n=271; 46.9% male; 67.3 yrs<br>Replication - SPIROMICS-Metabolon: n=445; 54.8% males; 65.3 yrs<br>Replication - SPIROMICS-UC: n=76; 52.6% male; 61.6 yrs	LC-MS (Metabolon)	Plasma	1-stearoyl-2-oleoyl-GPE (18:0/18:1); propionylcarnitine (C3); ergothioneine; 3-formylindole; 1-stearoyl-2-linoleoyl-GPE (18:0/18:2)*; glutamate; 1-stearoyl-2-docosahexaenoyl-GPE (18:0/22:6)*; palmitoyl-linoleoyl-glycerol (16:0/18:2) [2]; formiminoglutamate; retinol (Vitamin A); choline; isovalerylcarnitine (C5); 1-carboxyethylleucine; 1-carboxyethylvaline; phosphocholine; valine; gamma-glutamylleucine; 1-palmitoyl-2-oleoyl-GPE (16:0/18:1); aspartate; cortolone glucuronide (1)	myristoleoylcarnitine (C14:1); decanoylcarnitine (C10); sphingomyelin (d18:2/18:1); cis-4-decenoylcarnitine (C10:1); laurylcarnitine (C12); 5-dodecenoylcarnitine (C12:1); N6-carboxymethyllysine; palmitoyl sphingomyelin (d18:1/16:0); octanoylcarnitine (C8); 1-(1-enyl-palmitoyl)-2-palmitoleoyl-GPC (P-16:0/16:1); palmitoyl-sphingosine-phosphoethanolamine (d18:1/16:0); palmitoleoylcarnitine (C16:1); glycosyl ceramide (d18:2/24:1, d18:1/24:2); glutamine; 3-methoxytyrosine; glycosyl-N-behenoyl-sphingadienine (d18:2/22:0); hypotaurine; 1-(1-enyl-palmitoyl)-2-oleoyl-GPC (P-16:0/18:1); sphingomyelin (d18:2/16:0, d18:1/16:1); suberoylcarnitine (C8-DC)	diacylglycerol and BCAA (leucine, isoleucine, and valine)
FEV1/FVC	Halper-Stromberg et al	Bronchoalveolar Lavage Fluid from COPD Patients Reveals More Compounds Associated with Disease than Matched Plasma	SPIROMICS cohort<br>12 Non-smokers/56 Smokers/47 COPD<br>50.7% male<br>56/58/64 (NS/Smokers/COPD)	LC-MS	BALF	Phosphatidylserine (37:3); Lophocerine; p-cresol; Phosphatidylethanolamine (38:3); Phosphatidycholine (40:6); Phosphatidycholine (40:6) (isomer); Phosphatidycholine (32:1); Glycocholic acid; Monogalactosyldiacylglycerol (36:5); S-(Phenylacetothiohydroximoyl)-L-cysteine; Sphingomyelin (d18:1/24:1); Phosphatidylethanolamine (35:1); N-palmitoyl glycine; L-Threonylcarbamoyladenylate; Decaprenyl phosphate; Mycalamide B; Phosphatidycholine (36:4); Phosphatidylethanolamine (36:3); Phosphatidycholine (34:2); Homocysteine; Sulfoquinovosyl monoacylglycerol (16:1); Phosphatidylethanolamine (34:2); cardiolipin (70:0); cardiolipin (72:7)	Ceramide (d18:1/16:0)	amino acid derived compounds; fatty acids; phospholipids (phosphatidylethanolamines, lysophosphatidylethanolamines, lysophosphatidylcholines, phosphatidylserines, phosphatidylinositols, phosphatidylcholines)

Outcome	Authors	Title	Sample Description	Analytic Platform	Sample Type	Positively Associated Metabolites	Negatively Associated Metabolites	Pathways Identified
FEV1/FVC	Halper-Stromberg et al	Bronchoalveolar Lavage Fluid from COPD Patients Reveals More Compounds Associated with Disease than Matched Plasma	SPIROMICS cohort 12 Non-smokers/56 Smokers/47 COPD 50.7% male 56/58/64 (NS/Smokers/COPD)	LC-MS	Plasma	arginine; isoleucine; serine		
FEV1 % predicted	Mastej et al	Identifying Protein–metabolite Networks Associated with COPD Phenotypes	COPDGene Cohort 426 Controls/478 COPD/92 PRISm 12 missing Spirometry 50.9% males 64.6/71.1/67.3/72 (HC/COPD/PRISm/MissSpiro)	LC-MS	Plasma	Phosphocholine; Ergothioneine	: 5-hydroxyhexanoate; Palmitoleoylcarnitine (C16:1); Myristoleoylcarnitine (C14:1); Cis-4-decenoylcarnitine (C10:1); (N(1) + N(8))-acetylspermidine	
FEV1 % predicted	Diao et al	Disruption of histidine and energy homeostasis in chronic obstructive pulmonary disease	79 COPD/59 smokers no COPD/7 non-smokers 100% male 58.8/56.8/57.4 yrs (COPD/Smoker/NS)	NMR	Fasting Serum and Plasma	creatine; histidine; threonine; lactate; proline; serine		
FEV1 % predicted	Labaki et al	Serum amino acid concentrations and clinical outcomes in smokers	SPIROMICS cohort: n=157; 49.7% male; 53.7 yrs	NMR	Serum	tryptophan		
FEV1 % predicted†	Gillenwater et al	Plasma Metabolomic Signatures of Chronic Obstructive Pulmonary Disease and the Impact of Genetic Variants on Phenotype-Driven Modules	Discovery - COPDGene Cohort: n=957; 51.2% male; 68.3 yrs Replication - COPDGene-Emory: n=271; 46.9% male; 67.3 yrs Replication - SPIROMICS-Metabolon: n=445; 54.8% males; 65.3 yrs Replication - SPIROMICS-UC: n=76; 52.6% male; 61.6 yrs	LC-MS (Metabolon)	Plasma	phosphocholine; ergothioneine; gamma-glutamyl-2-aminobutyrate; dehydroepiandrosterone sulfate (DHEA-S); 3-formylindole; androstenediol (3beta,17beta) monosulfate (1); retinol (Vitamin A); cortisone; 1-stearoyl-2-oleoyl-GPE (18:0/18:1); iminodiacetate (IDA); androstenediol (3beta,17beta) monosulfate (2); branched chain 14:0 dicarboxylic acid**; asparagine; 1-stearoyl-2-oleoyl-GPI (18:0/18:1)*; 2-aminobutyrate; alanine; beta-cryptoxanthin; 1-stearoyl-2-linoleoyl-GPI (18:0/18:2); 3-methyl catechol sulfate (2); 1,2-dilinoleoyl-GPC (18:2/18:2)	N6-carboxymethyllysine; cis-4-decenoylcarnitine (C10:1); 5-dodecenoylcarnitine (C12:1); C-glycosyltryptophan; myristoleoylcarnitine (C14:1)*; X - 13553; vanillylmandelate (VMA); (N(1) + N(8))-acetylspermidine; palmitoleoylcarnitine (C16:1)*; adipoylcarnitine (C6-DC); decanoylcarnitine (C10); X - 12026; suberoylcarnitine (C8-DC); octanoylcarnitine (C8); mannose; laurylcarnitine (C12); N2,N2-dimethylguanosine; nonanoylcarnitine (C9); glutamine conjugate of C6H10O2 (2)*	

Outcome	Authors	Title	Sample Description	Analytic Platform	Sample Type	Positively Associated Metabolites	Negatively Associated Metabolites	Pathways Identified
FEV1 % predicted	Balgoma et al	Linoleic acid-derived lipid mediators increase in a female-dominated subphenotype of COPD	Karolinska COSMIC 25 COPD Smokers/10 COPD Former Smoker/40 Smokers/39 Never Smokers 50% males 59.5/60/54/56.5 (COPD S/COPD FS/S/NS)	LC-MS/MS	BALF	*Females:* EpOMEs; DiHOMEs.	*Score*: 9,10,13-TriHOME (9,10,13-trihydroxy-11E-octadecenoic acid), 12(13)-EpOME (12[13]epoxy-9Z-octadecenoic acid), 9(10)-EpOME (9[10]-epoxy-12Z-octadecenoic acid), 9,10-DiHOME (9[10]-dihydroxy-12Z-octadecenoic acid), 12,13-DiHOME (12[13]-dihydroxy-12Zoctadecenoic acid), 12-HHTrE (12-hydroxy-5Z,8E,10E-heptadecatrienoic acid), 5-KETE (5-oxo-ETE, 5-oxo- 6E,8Z,11Z,14Z-eicosatetraenoic acid), TXB2 (thromboxane B2) and 9-KODE (9-oxo-10E,12Z-octadecadienoic acid)	
FEV1 % predicted	Balgoma et al	Linoleic acid-derived lipid mediators increase in a female-dominated subphenotype of COPD	Karolinska COSMIC 25 COPD Smokers/10 COPD Former Smoker/40 Smokers/39 Never Smokers 50% males 59.5/60/54/56.5 (COPD S/COPD FS/S/NS)	LC-MS/MS	BALF	PGF2α, 12-HHTrE, 12-HETE, 11(12)-EpETrE, 9,10,13-TriHOME, 5(6)-EpETrE, 11-β-PGF2α	5,6-DiHETrE, 5-HEPE, 5-HETE	
FEV1	McClay et al	1H Nuclear Magnetic Resonance Metabolomics Analysis Identifies Novel Urinary Biomarkers for Lung Function	197 COPD/90 Smokers/105 Never Smokers 56.4% males 64.7/57.2/56.5 (COPD/Smoker/NS)	NMR	Urine	trigonelline; hippurate; formate		
Lung Function	Xue et al	Metabolomic profiling of anaerobic and aerobic energy metabolic pathways in chronic obstructive pulmonary disease	140 COPD/20 HC 78.8% males 60/52 yrs (COPD/HC)	UHPLC-Q-TOF/MS	Fasting Serum	Citrate; alpha-ketogluatara; Succinate; Fumarate; Oxa	Isocitrate; Malate; Pyruvic; Lactic	
FEV1	Yu et al	Metabolomics Identifies Novel Blood Biomarkers of Pulmonary Function and COPD in the General Population	ARIC Cohort (n= 2,354 African Americans, 1529 European American); 39.8% male; 53.0/54.6 yrs (AA/EA) KORA cohort (n=859 Europeans); 46.8% male; 53.8 yrs	LC-MS	Serum	glycine; 3-phenylpropionate (hydrocinnamate); asparagine; glutamine; serotonin (5HT); glycerate; gamma-glutamylthreonine; gamma-glutamylleucine; gamma-glutamylvaline	3-(4-hydroxyphenyl)lactate; 2-methylbutyrylcarnitine (C5); alpha-hydroxyisovalerate; isoleucine; lactate; fructose; mannose; glycerol; 7-alpha-hydroxy-3-oxo-4-cholestenoate (7-Hoca); N2,N2-dimethylguanosine; pseudouridine	Aminoacyl-tRNA biosynthesis; Phenylalanine metabolism; Nitrogen metabolism; Alanine, aspartate and glutamate metabolism
FVC	Yu, Bing	Metabolomics Identifies Novel Blood Biomarkers of Pulmonary Function	ARIC Cohort (n= 2,354 African Americans, 1529 European American); 39.8%	LC-MS	Serum	glycine; N-acetylglycine; asparagine; glutamine; 3-phenylpropionate (hydrocinnamate); 5-oxoproline; biliverdin	isoleucine; 2-methylbutyrylcarnitine (C5); 3-(4-hydroxyphenyl)lactate; tyrosine; valine; phenylalanine; mannose; lactate; fructose; glucose; glycerol; pseudouridine; urate	Aminoacyl-tRNA biosynthesis; Phenylalanine metabolism

Outcome	Authors	Title	Sample Description	Analytic Platform	Sample Type	Positively Associated Metabolites	Negatively Associated Metabolites	Pathways Identified
		and COPD in the General Population	male; 53.0/54.6 yrs (AA/EA) KORA cohort (n=859 Europeans); 46.8% male; 53.8 yrs					
Exacerbation vs Not	Celejewska-Wójcik et al.	Eicosanoids and Eosinophilic Inflammation of Airways in Stable COPD	76 COPD/37 HC 68% male 65 yrs (mean age)	GC-MS HPLC-MS	Sputum	PGD2; 12-oxo-ETE; 5-oxo-ETE		
Exacerbation vs Not	Labaki et al	Serum amino acid concentrations and clinical outcomes in smokers	SPIROMICS cohort: n=157; 49.7% male; 53.7 yrs	NMR	Serum		O-acetylcarnitine; Lysine; 2-hydroxybutyrate; Tryptophan; Leucine; Ornithine; Valine; Choline; Serine; Glutamine; Isoleucine; Histidine; Betaine; Proline; Phenylalanine; Creatine; Carnitine; Threonine; Tyrosine; Glucose; Creatinine; Citrate; Alanine; Methionine; Glycine; Lactate; Glutamate	
Exacerbations	Esther et al	Identification of Sputum Biomarkers Predictive of Pulmonary Exacerbations in Chronic Obstructive Pulmonary Disease	SPIROMICS cohort 77 healthy non-smokers /341 smokers perserved spirometry/562 COPD 53% male 55.4/59.6/65 yrs (HC/Smokers/COPD)	UPLC-MS	Sputum	Sialic Acid; Hypoxanthine		
Exacerbation Frequency	Cruickshank-Quinn et al.	Metabolomics and transcriptomics pathway approach reveals outcome-specific perturbations in COPD	COPDGene Cohort: (n=149); 53.0% male; 63.1 yrs	LC-MS	Plasma	Carnitine (C14:2)	L-Glutamine; Pyroglutamic acid; Tryptophan; Tyrosine; Oleamide; Dimethylallyl diphosphate; Leupeptin	Aminoacyl-tRNA biosynthesis; Antigen processing and presentation; Glycerophospholipid metabolism; Mineral absorption; Protein digestion and absorption; Ribosome; RNA transport
Exacerbation Frequency	Gillenwater et al	Plasma Metabolomic Signatures of Chronic Obstructive Pulmonary Disease and the Impact of Genetic Variants on Phenotype-Driven Modules	Discovery - COPDGene Cohort: n=957; 51.2% male; 68.3 yrs Replication - COPDGene-Emory: n=271; 46.9% male; 67.3 yrs Replication - SPIROMICS-Metabolon: n=445; 54.8% males; 65.3 yrs Replication -	LC-MS (Metabolon)	Plasma		N,N,N-trimethyl-alanylproline betaine (TMAP)	

Outcome	Authors	Title	Sample Description	Analytic Platform	Sample Type	Positively Associated Metabolites	Negatively Associated Metabolites	Pathways Identified
			SPIROMICS-UC: n=76; 52.6% male; 61.6 yrs					
Exacerbation Severity	Cruickshank-Quinn et al.	Metabolomics and transcriptomics pathway approach reveals outcome-specific perturbations in COPD	COPDGene Cohort: (n=149); 53.0% male; 63.1 yrs	LC-MS	Plasma	Lysine; Cholic acid; Alpha-D-glucose; Mannitol	Acetylcarnitine; Citrulline; Creatinine; L-Glutamine; L-Norvaline; 7-Hydroxy-3-oxocholanoic acid; 1,6-Anhydro-β-D-glucopyranose; 2-Deoxy-D-glucose; 3-Methyladenine; Hypoxanthine	ABC transporters; Aminoacyl-tRNA biosynthesis; Arginine and proline metabolism; Arginine biosynthesis; Autophagy; Glycerophospholipid metabolism; Glycine, serine and threonine metabolism; Insulin resistance; Mineral absorption; Phenylalanine, tyrosine and tryptophan biosynthesis; Protein digestion and absorption; Purine Metabolism; Retrograde endocannabinoid signaling; Sphingolipid metabolism; Sphingolipid signaling pathway
Exacerbations (Moderate & Severe)	Bowler et al	Plasma Sphingolipids Associated with Chronic Obstructive Pulmonary Disease Phenotypes	COPDGene Cohort *Targeted*: (**n**=129; 57% male; 63 yrs) *Untargeted*: (**n**=131; 56% male; 64 yrs)	LC-MS	Plasma	Trihexosylceramide (d18:1/16:0); 3-O-Sulfogalactosylceramide (d18:1/16:0); Galabiosylceramide (d18:1/24:1(15Z))	Sphingosine 1-phosphate	
GOLD Stage	Gillenwater et al	Plasma Metabolomic Signatures of Chronic Obstructive Pulmonary Disease and the Impact of Genetic Variants on Phenotype-Driven Modules	Discovery - COPDGene Cohort: n=957; 51.2% male; 68.3 yrs Replication - COPDGene-Emory: n=271; 46.9% male; 67.3 yrs Replication - SPIROMICS-Metabolon: n=445; 54.8% males; 65.3 yrs Replication - SPIROMICS-UC: n=76; 52.6% male; 61.6 yrs	LC-MS (Metabolon)	Plasma		Ergothioneine	
IL6	Diao et al	Disruption of histidine and energy homeostasis in chronic obstructive pulmonary disease	79 COPD/59 smokers no COPD/7 non-smokers 100% male 58.8/56.8/57.4 yrs (COPD/Smoker/NS)	NMR	Fasting Serum and Plasma	creatine; glycine; histidine; carnitine; lactate; lysine; phenylalanine; serine; tyrosine		

Outcome	Authors	Title	Sample Description	Analytic Platform	Sample Type	Positively Associated Metabolites	Negatively Associated Metabolites	Pathways Identified
TNF-alpha	Diao et al	Disruption of histidine and energy homeostasis in chronic obstructive pulmonary disease	79 COPD/59 smokers no COPD/7 non-smokers 100% male 58.8/56.8/57.4 yrs (COPD/Smoker/NS)	NMR	Fasting Serum and Plasma	histidine; betaine; glutamine; acetylcarnitine; valine		
Subtyping	Gillenwater et al	Multi-omics subtyping pipeline for chronic obstructive pulmonary disease	COPDGene Cohort: n=1057; 50.5% male; 67.6 yrs	LC-MS (Metabolon)	Plasma	Support Vector Machine with Recursive Feature Extraction Metabolites: dehydroisoandrosterone sulfate (DHEA-S); 3-(3-amino-3-carboxypropyl)uridine; X– 12117 ; stearoyl sphingomyelin (d18:1/18:0); hydroxy-CMPF; N6-carbamoylthreonyladenosine; N-formylmethionine; sphingomyelin (d18:1/20:1, d18:2/20:0); sphingomyelin ; (d18:1/17:0, d17:1/18:0, d19:1/16:0); 1-palmitoyl-2-linoleoyl-GPC (16:0/18:2); 3-carboxy-4-methyl-5-propyl-2-furanpropanoate (CMPF); pyroglutamine		Sphingomyelins
6MWD	Labaki et al	Serum amino acid concentrations and clinical outcomes in smokers	SPIROMICS cohort: n=157; 49.7% male; 53.7 yrs	NMR	Serum	tryptophan		

AA - African American; AECOPD - Acute Exacerbation of COPD; BALF - bronchoaveolar lavage fluid; Cach - cachexia; Cer - ceramide; DI-ESI-QTOF-MS - direct infusion electrospray ionization triple quadrupole time-offlight mass spectrometry; EA - European American; EBC – exhaled breath condensate; Emph - emphysematous; ESI-MS - electrospray ionization - MS; FS - former smoker; GC - gas chromatography; GC-IMS - gas chromatography - ion mobility spectrometry; HC – healthy controls; HDL - high density lipoprotein; HPLC-MS - high-performance liquid chromatography/tandem mass spectrometry; HS - Healthy smoker; ILD - Interstitial lung disease; IMS- ion mobiity spectrometry; IMS-MCC - Ion Mobility Spectrometer - Multi-Capillary Column; LC - liquid chromatography; LC - lung cancer; LC-QTOF-MS - LC-Quadrupole Time-of-Flight-MS; LDL - low density lipoprotein; LTD4 - leukotriene D4; LTE4 - leukotriene E4; missSpiro - missing spirometry measure; MS - mass spectrometry; NMR – nuclear magnetic resonance; non-E - non-emphysematous; Non-S - Non-smokers; NS - never-smoker; PGD2 - prostaglandin D2; PGE2 - prostaglandin E2; PI - phosphoinositol; PRISm - preserved Ratio Impaired Spirometry; S - smoker; S-COPD - Stable COPD; SESI-HRMS - secondary electrospray ionization – high-resolution MS; TB - tuberculosis-related COPD; TD-GC-APCI_MS - thermal desorption-GC- atmospheric pressure chemical ionization - MS; UHPLC-Q-TOF/MS - ultra-high performance liquid phase series quadrupole flight-time/secondary MS; UPLC-MS - ultra-performance liquid chromatography MS; VLDL - very low density lipoprotein; yrs -year
